# Elucidating the developmental dynamics of mouse stromal cells at single-cell level

**DOI:** 10.1093/lifemedi/lnac037

**Published:** 2022-09-05

**Authors:** Xing Fang, Mengmeng Jiang, Mingyong Zhou, Jikai Shao, Xiunan Fang, Jingjing Wang, Yuting Fu, Yuan Liao, Haide Chen, Renying Wang, Lijiang Fei, Huiyu Sun, Peijing Zhang, Jingang Huang, Xiaoping Han, Guoji Guo

**Affiliations:** Center for Stem Cell and Regenerative Medicine, Bone Marrow Transplantation Center of the First Affiliated Hospital, Zhejiang University School of Medicine, Hangzhou, Zhejiang 310000, China; Zhejiang Provincial Key Lab for Tissue Engineering and Regenerative Medicine, Dr. Li Dak Sum & Yip Yio Chin Center for Stem Cell and Regenerative Medicine, Hangzhou 310058, China; Liangzhu Laboratory, Zhejiang University Medical Center, Hangzhou 311121, China; Center for Stem Cell and Regenerative Medicine, Bone Marrow Transplantation Center of the First Affiliated Hospital, Zhejiang University School of Medicine, Hangzhou, Zhejiang 310000, China; Zhejiang Provincial Key Lab for Tissue Engineering and Regenerative Medicine, Dr. Li Dak Sum & Yip Yio Chin Center for Stem Cell and Regenerative Medicine, Hangzhou 310058, China; Center for Stem Cell and Regenerative Medicine, Bone Marrow Transplantation Center of the First Affiliated Hospital, Zhejiang University School of Medicine, Hangzhou, Zhejiang 310000, China; Liangzhu Laboratory, Zhejiang University Medical Center, Hangzhou 311121, China; Liangzhu Laboratory, Zhejiang University Medical Center, Hangzhou 311121, China; Center for Stem Cell and Regenerative Medicine, Bone Marrow Transplantation Center of the First Affiliated Hospital, Zhejiang University School of Medicine, Hangzhou, Zhejiang 310000, China; Center for Stem Cell and Regenerative Medicine, Bone Marrow Transplantation Center of the First Affiliated Hospital, Zhejiang University School of Medicine, Hangzhou, Zhejiang 310000, China; Zhejiang Provincial Key Lab for Tissue Engineering and Regenerative Medicine, Dr. Li Dak Sum & Yip Yio Chin Center for Stem Cell and Regenerative Medicine, Hangzhou 310058, China; Liangzhu Laboratory, Zhejiang University Medical Center, Hangzhou 311121, China; Center for Stem Cell and Regenerative Medicine, Bone Marrow Transplantation Center of the First Affiliated Hospital, Zhejiang University School of Medicine, Hangzhou, Zhejiang 310000, China; Center for Stem Cell and Regenerative Medicine, Bone Marrow Transplantation Center of the First Affiliated Hospital, Zhejiang University School of Medicine, Hangzhou, Zhejiang 310000, China; Center for Stem Cell and Regenerative Medicine, Bone Marrow Transplantation Center of the First Affiliated Hospital, Zhejiang University School of Medicine, Hangzhou, Zhejiang 310000, China; Liangzhu Laboratory, Zhejiang University Medical Center, Hangzhou 311121, China; Medical Research Center, Guangdong Provincial Key Laboratory of Malignant Tumor Epigenetics and Gene Regulation, Guangdong-Hong Kong Joint Laboratory for RNA Medicine, Sun Yat-Sen Memorial Hospital, Sun Yat-Sen University, Guangzhou 510120, China; Center for Stem Cell and Regenerative Medicine, Bone Marrow Transplantation Center of the First Affiliated Hospital, Zhejiang University School of Medicine, Hangzhou, Zhejiang 310000, China; Center for Stem Cell and Regenerative Medicine, Bone Marrow Transplantation Center of the First Affiliated Hospital, Zhejiang University School of Medicine, Hangzhou, Zhejiang 310000, China; Liangzhu Laboratory, Zhejiang University Medical Center, Hangzhou 311121, China; Zhejiang University-University of Edinburgh Institute, Zhejiang University School of Medicine, Zhejiang University, Hangzhou 314400, China; Zhejiang Provincial Key Lab for Tissue Engineering and Regenerative Medicine, Dr. Li Dak Sum & Yip Yio Chin Center for Stem Cell and Regenerative Medicine, Hangzhou 310058, China; Institute of Hematology, Zhejiang University, Hangzhou 310058, China

Dear Editor,

Stromal cells cooperating with extracellular matrix (ECM) in tissues are essential support components for many functional cells such as epithelial, endothelial, muscle, and neuronal cells. Single-cell atlases of individual tissues and organism have described the heterogeneity of stromal cells [1]. With previous studies mainly focused on adult stage or disease state, the characterization of stromal cells in mammal development still remains unclear [2]. Here, we constructed a detailed development-related transcriptional profiles of stromal cells by collecting single-cell data from embryo to adult and taking advantage of our previous works of mouse cell atlas, human cell landscape, and recent mouse cell differentiation atlas [1, 3, 4]. Analysis of whole procedure of embryo development and mature revealed strong temporal heterogeneity of stromal cells with obvious stage-specific molecular dynamics.

Since precise annotation of each cell type is a key element for downstream scRNA-seq analysis, six large-scale single-cell atlases were collected and compared first, half of which were generated by our microwell-seq platform and the remaining half from 10× Genomics (Figs. 1A and S1A). Annotations among different single-cell atlases were compared, showing overall good correlation with AUROC >90%, as shown in the representative tissues of heart and kidney (Figs. S1B and S1C). We then compared the annotation across five growth stages, and observed high consistency of main cell types in mouse development, such as heart and lung tissues (Figs. S1D and S1E). Hierarchy of cell types of individual tissue with marker genes was summarized and categorized in Table S1. Though almost all tissues contained immune, endothelial, stromal, and epithelial lineages, tissues with different germ layers tended to have some bias in cell type composition based on their origin (Fig. 1B; Table S1). Among these lineages, stroma showed widespread diversity across tissues and developmental stages.

**Figure 1.**
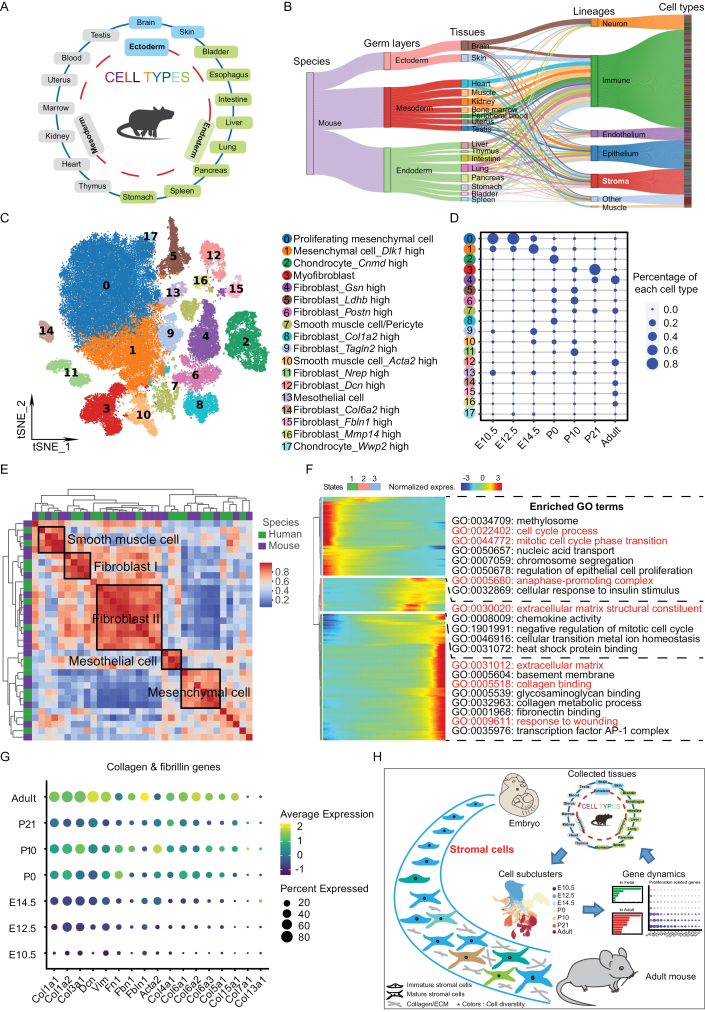
Dynamic change of stromal cells in mouse development. (A) Illustration of study content. (B) Sankey diagrams showing hierarchy of cell types of major mouse tissues summarized from different single-cell databases. (C) *t*-SNE plot of all stromal cells at seven different time points during mouse development. Cells are colored by cell-type cluster. (D) Relative abundance of each cell type in developmental mSCL. The numbers represent cell cell-type cluster corresponding to Fig. 1C. The sizes of bubbles indicate the contributions of cells from each stage to a cluster. (E) Similarity of stromal cell types between human and mouse. AUROC scores were used to measure the similarity of cell types: red represents high correlation, blue represents low correlation. Species information is marked by different colors. (F) Heatmap showing the gene expression dynamics of stromal cells during mouse development. Genes (row) are clustered and cells (column) are ordered according to the pseudotime trajectory. The top 300 genes in each cluster were analyzed with GO, and represent GO terms are displayed. The colored texts indicate focused GO terms. (G) Expression of collagen and fibrillin genes across seven developmental stages. Circle sizes denote percentages of cells from each stage, and color encodes average expression across all cells within stages. (H) Graphical summary. A developmental mSCL was constructed by leveraging published single-cell databases. Both tissue- and stage-specificity of stromal cells were gradually generated during mouse development. Cell proliferation in embryonic stage and collagen accumulation in adult were two remarkable features in life cycle of stromal cells.

To explore these stromal cells, we constructed a mouse stromal cell landscape (mSCL) spanning developmental stages of embryonic day, postnatal day, and adult by integrating stromal cells extracted from individual tissue (Figs. 1C and S2A–D). Eighteen clusters were identified with major stromal cell types of fibroblasts (*Col1a2*, *Mfap5*), smooth muscle cell (*Acta2*, *Myh11*), mesothelial cell (*Upk3b*, *Lrrn4*), and chondrocyte (*Col9a1*, *Sox9*) (Fig. S2E; Table S2). Among the seven developmental stages, heterogeneity of stromal cells in embryonic stages was smaller than that in adult (Fig. 1D). For instance, only proliferating mesenchymal cells and *Dlk1*+ mesenchymal cells specifically existed in embryonic stages, while *Dcn*+, *Col6a2*+, *Fbln1*+, and *Mmp14*+ fibroblasts were enriched in adult stage. GO analysis for mesenchymal cells showed enrichment of cytosolic ribosome and mitochondrial respirasome, which linked to the rapid cell proliferation at embryonic stages (Fig. S3A). Notably, there were lots of tissue-specific fibroblast cells existed in developmental process (Figs. S2F and S3B–D; Table S2). Myofibroblast (placenta, ovary, and uterus) with marker genes *Hspa1b*, *Bag3,* and *Dnajb1*, were enriched terms of response to unfolded protein, positive regulation of programmed cell death, and regulation of angiogenesis, which were associated with stress response to environmental stimulus post birth. *Postn*+ fibroblast (heart and liver) with highly expressed *Aspn* and *Ogn* genes, were enriched in ECM, ossification, and skeletal system development, in accordance with structural development of heart valves and hepatobiliary duct [5]. *Nrep*+ fibroblast (lung) with *Tnc* and *Adh1* were reported contributing to postnatal lung development by shaping alveolar septa, which were associated with GO terms of structural molecule activity, elastic fiber, and regulation of cell growth [6]. Besides, mSCL showed high correlation with human stromal cell landscape in main cell types, such as fibroblast, smooth muscle cell, and mesothelial cell (Figs. 1E and S4A–E; Tables S3 and S4). Especially, the mesenchymal cell in embryonic stages also exhibited high correlation between human and mouse with high level of *Cdk4*, *Stmn1*, and *Dlk1*, etc., which meant high developmental conservation of stromal cells (Tables S2 and S3). These results described both tissue- and stage-specificity of stromal cells in mouse development, and high conservation of these cell types in species evolution between human and mouse. Further analysis of secreted profiles showed that relative low expression of collagen and ECM-related genes in the two mesenchymal cells (embryo tissues) while different combinatorial expression of these genes in other stromal cell types corresponding to each tissue (Figs. S3E–G). And differential gene expression analysis between stromal cells from these 18 tissues revealed several canonical and previously undescribed tissue-enriched genes (Fig. S3H). We thus speculated the diverse stromal cell types with tissue specificity formed different organizational microenvironment, and promoted comprehensive tissue formation in the development of multiorgan species.

We further analyzed the cell trajectory and gene signature of these stromal cells. Uniform Manifold Approximation and Projection showed the clear cell fate transition of stromal cells during mouse development (Figs. S5A and S5B). Trajectory reconstruction by Monocle2 confirmed continuous the cell fate transition from embryonic to adult stage (Figs. S5C–E). Coupled with Gene Ontology (GO) annotation analysis, the up-regulated genes in early stages enriched function of cell cycle process, nucleic acid transport, and cellular response to insulin stimulus, following with enriched ECM structure and negative regulation of cell cycle, finally the enrichment of fibronectin binding and response to wounding at terminal stages (Fig. 1F). Similar cell differentiation pattern of stromal cells was observed in development of individual tissue, e.g. heart (Figs. S5F and S5G). Cell proliferation and collagen change were shown as two important features in stromal cells. Further analysis exhibited most of cell proliferation genes (such as *Cdk4*, *Stmn1*, and *Ccnb1*) were decreasingly expressed during mouse development, while both collagen plus fibrillin genes (such as *Col1a1*, *Col3a1,* and *Fbn1*) and ECM-remodeling genes were gradually increased (Figs. 1G, S6A and S6B). Taken together, we hypothesized that stromal cells mainly presented a strong proliferating tendency to amplify cell number in early stage; then showed an upregulation of stroma-related genes in development procedure, which facilitated stabilizing their cell fate gradually; and eventually arrived a steady cell state in adult.

In addition, we noticed some fetal-specific genes (such as imprinted genes *H19*, *Dlk1*, *Cdkn1c*, *Mest*, and other gene *Crabp1*) were only highly expressed in mouse embryonic and neonatal stages, among which *Dlk1* was mainly expressed in stromal cells and a few of mesoderm derived progenitor cells (Figs. S6C and S6D). These fetal genes also showed similarly fetal-specific high-expression pattern in human stromal cells and were closely linked to normal embryo development and body growth (Figs. S6E and S6F). Meanwhile, some of these genes (*Dlk1*, *H19*, and *Crabp1*) were reported showing special expression in diseases, which inspired us to explore the potential relationship between these fetal genes and diseases [7]. *Lrrc15*+ cluster was identified as a perturbation-specific fibroblast in the reported fibroblast atlas, including fibroblasts under diverse perturbed-states of infection, injury, cancer, fibrosis, metabolic changes, and arthritis [2]. Our further analysis showed most of these fetal-specific genes were activated in the *Lrrc15*+ cluster of mouse perturbed-state atlas but not in steady-state atlas (Figs. S6G and S6H). To explore the relationship between the *Lrrc15*+ cluster and stromal cells in development, we reclustered cells in *Lrrc15*+ cluster and mapped to mSCL. Interestingly, a subset (Lrrc15_#0) in *Lrrc15*+ cluster with enrichment of fetal genes was closely correlated with the *Col1a2*+ fibroblast of developing P0 stages in mSCL while the remaining were correlated to stromal cell types in adult stage (Figs. S7A–H). The Lrrc15_#0 subset was mainly originated from the perturbed state of skin wound, and a previous report proved that regeneration-competent fibroblasts with stemness existed in skin large wound (Figs. S7B and S7C) [8]. We therefore inferred fetal-specific genes might play important role in early stromal cell development, and their reactivation in adult would reprogram stromal cell close to a fetal state.

In summary, we constructed a developmental mSCL by leveraging the published single-cell databases (Fig. 1H). Our analysis suggested cell proliferation in embryonic stage and collagen accumulation in adult were two remarkable features in life cycle of stromal cells. Contrary to high specialization in adult, stromal cells in early stage presented a strong proliferating tendency with low heterogeneity, which also implied these cells might maintain a stemness state. Also, imprinted genes such as *Dlk1*, *H9*, and *Cdkn1c*, were also highlighted as related in early stromal cells by our work. Associated the strong proliferation ability of early stromal cells and these genes’ role in tissue progenitors, we speculated the imprinted genes could be involved in the proliferation or stemness maintenance of stromal cells [9]. The cell fate decision of imprinted genes could also partly explain the phenomenon of reprograming stromal cell by these genes in some disease states. During the maturing process of stromal cells, collagen, and ECM-related genes showed progressive upregulation. Interesting, during mouse aging, these stromal-related genes were reported obviously down-regulated in some tissues [10]. These results suggested stromal-associated genes arrived a peak expression in mouse adult stage, and were related to the steady-state maintenance of stromal cells. Our work provided a systematical way to analyze the developmental transcriptomic dynamics of stromal cells, which could be broadened to other widespread cell types in future.

## Research limitations

Our study systematically illustrated the tissue- and stage-heterogeneity of stromal cells in mouse development. However, the real spatial distribution of these stromal cell is still lack. Cell positional information can be used to infer cell communication between stromal cell with other cell types and cell migration during development, which help understand function of these identified cell types with a cellular network perspective.

## Supplementary Material

lnac037_suppl_Supplementary_Figures

lnac037_suppl_Supplementary_Table_S1

lnac037_suppl_Supplementary_Table_S2

lnac037_suppl_Supplementary_Table_S3

lnac037_suppl_Supplementary_Table_S4
