# Supplementary material for: Elucidating the developmental dynamics of mouse stromal cells at single-cell level
Source: Life Med. 2022 Sep 5;1(1):45–8. doi: 10.1093/lifemedi/lnac037 (PMC11749665; doi:10.1093/lifemedi/lnac037)
Supplement: lnac037_suppl_Supplementary_Figures [file lnac037_suppl_Supplementary_Figures.pdf]

## SUPPLEMENTARY INFORMATION

### Method Details

#### Integration and classification of scRNA-seq data

We mainly collected the published large-scale single cell atlases, including three versions from haman and three from mouse. The collected information contained annotation information of cell types and the processed data (Seurat or scanpy object) in each tissue if existed[1,2]. For the tissues that existed in more than one databases, we firstly assigned the cell annotation information to the processed data, and conducted comparative analysis of different data by MetaNeighbor tool[3]. The annotation correlation of cell types in each tissue were compared between platforms of microwell-seq and 10×Genomics, between species of human and mouse, and among developmental stages of mouse. Referred to compared results, cell types of individual tissue were further classified into sub-clusters by leveraging the top-level markers, and the used marker genes were list under cell types for reference. Finally, the sub-clusters were classified into several major cell lineages including epithelial, endothelial, stromal, immune, muscle, neuron, et al. Upon these works, we summarized all the detected cell types with hierarchical organization for main tissues across the large-scale single cell databases. The detail information was listed in Table S1.

#### Construction of stromal cell landscapes of mouse and human

Data of MCDA had the widespread cell types and more developmental stages of mouse, we mainly adopted it as following analysis. We obtained all single-cell data of multiple tissues from embryonic to adult stage including E10.5, E12.5, E14.5, P0, P10, P21, and adult (E: embryonic day, P: postnatal day). To ensure comparability, single-cell data from every individual tissue was filtered, clustered, and annotated by Seurat single-cell analysis pipeline (v3.2.2) as our published work[1,4]. Following information of supplementary table 1, all cell types of stroma in the cell lineages for each tissue were extracted separately and merged together. To filter merged data with seurat, we adopted the top 21 PCA dimensions, set the resolution for clusters and runtsne to 0.8, and kept other arguments defaults[5]. All clusters were annotated based on the top-level markers

and corresponding tissue information. Non-stromal cells were removed, and the remained cells were clustered again with resolution value of 0.2 to obtain the stromal cell landscape. The lower resolution resulted less cell clusters facilitating investigation of common features among different conditions[6]. Due to the little batch effects of microwell-seq and unified experimental process of each tissue, we didn't use integrated method in this procedure[4]. To determine marker genes of each cluster or differentially expressed genes of different tissues, we used the Seurat function FindAllMarkers with default parameters. For human, we obtained all single-cell data of multiple tissues within fetal and adult stages in HCL data, and the detail processes for stromal cell landscape were similar to mouse.

**Cross-species stromal cell landscape analysis**

To compare the cell types from both species, we downloaded the homology correspondences between human and mouse provided by dmod-ENCODE[7]. The gene expression profiles for human and mouse were transformed into a corresponding format, merged together and normalized to the total number of transcripts and multiplied by 100,000. The formatted data were directly imported into MetaNeighbor tools under Python 3.6.5, analyzing through neighbor voting based on the spearman correlation between all human and mouse cells[8-10]. Then mean AUROC scores were obtained from MetaNeighbor and plot in R. To comparing cell types from two scRNA-seq platforms and multiple stages, MetaNeighbor were performed the same as above, except no need for genes transformation between two species.

**Trajectory inference and molecular dynamic analysis by Monocle2**

Cells in mouse stromal cell landscape, randomly sampled 300 single cells for each cell type without replacement (cell types fewer than 300 cells without sampling), were imported into Monocle2 R package (version 2.10.1)[11]. Single cell trajectory analysis was performed as the default arguments. The actual stage information of each cell types informed us of the start point of the pseudo-time in the first round of 'orderCells'. We then set this state as the root_state argument and called 'orderCells' again. 'DDRTree' was applied to reduce dimensions and the visualization functions 'plot_cell_trajectory' or 'plot_complex_cell_trajectory' were used to plot the minimum spanning tree on cells.

Then, we used the differential GeneTest function to find genes that had an expression pattern that varied according to pseudotime. The top 300 most significantly differentially expressed genes were used for heatmap show. Last, GO enrichment analyses were performed using Metascape[12]. Also, stromal cell of individual tissues, for example heart, were extracted in five developmental stages and analyzed as the same procedures.

**Comparison of fibroblast in perturb-state atlas to stromal cell landscape**

To better compare the cell state of perturb-state fibroblast in diseases with stromal cells in mouse development, we built a stromal cell mapping pipeline as our published works[6,9]. We used stromal cell clusters in each individual tissue, combined information of the cell-type, tissue and stage for each meta-cluster, and generated a total of 74 cell-type clusters. For each cell-type cluster, we randomly sampled 100 single cells without replacement (all cells for clusters with fewer than 100 cells), calculated the average expression, and repeated for three times. Then, we performed differential gene expression analysis for each cell type against all other cell types and selected the top 20 genes for each cell type. All selected genes were merged to create a combined feature gene list. This resulted in main transcriptome references for following analysis. For perturb-state fibroblast comparison, we randomly sampled 5000 single cells in *Lrrc15*+ cell type against each cell type within the prepared reference, and the Pearson correlations of given cell were calculated using the combined feature gene list. Only the top 1 mapping hit was shown for each data point.

Considering the heterogeneity of *Lrrc15*+ cluster in perturbed-state fibroblast atlases, we performed re-clustering analysis following the procedures of constructing perturbed-state atlases[6]. *Lrrc15*+ clusters were extracted, integrated with each tissue using Harmony[13]. We then provided the top 8 harmony dimensions as an input for UMAP and visualized the first two UMAP dimensions at a clustering resolution of 0.1. Four clusters were identified and annotated. To further compare these subclusters with cells in our mSCLs, we merged the two data, normalized, and integrated with Harmony. Cell information of previous annotation and stages were assigned separately on UMAP to visualize the final result.

## Method References


1. Stuart, T. *et al.* Comprehensive Integration of Single-Cell Data. *Cell* **177**, 1888-1902 e1821, (2019).
2. Wolf, F. A., Angerer, P. & Theis, F. J. SCANPY: large-scale single-cell gene expression data analysis. *Genome Biol* **19**, 15, (2018).
3. Crow, M., Paul, A., Ballouz, S., Huang, Z. J. & Gillis, J. Characterizing the replicability of cell types defined by single cell RNA-sequencing data using MetaNeighbor. *Nature communications* **9**, 884, (2018).
4. Han, X. *et al.* Mapping the Mouse Cell Atlas by Microwell-Seq. *Cell* **172**, 1091-1107 e1017, (2018).
5. Kalucka, J. *et al.* Single-Cell Transcriptome Atlas of Murine Endothelial Cells. *Cell* **180**, 764-779 e720, (2020).
6. Buechler, M. B. *et al.* Cross-tissue organization of the fibroblast lineage. *Nature*, (2021).
7. Celniker, S. E. *et al.* Unlocking the secrets of the genome. *Nature* **459**, 927-930, (2009).
8. Fischer, S., Crow, M., Harris, B. D. & Gillis, J. Scaling up reproducible research for single-cell transcriptomics using MetaNeighbor. *Nature protocols* **16**, 4031-4067, (2021).
9. Han, X. *et al.* Construction of a human cell landscape at single-cell level. *Nature* **581**, 303-309, (2020).
10. Wang, J. *et al.* Tracing cell-type evolution by cross-species comparison of cell atlases. *Cell Rep* **34**, 108803, (2021).
11. Qiu, X. *et al.* Reversed graph embedding resolves complex single-cell trajectories. *Nat Methods* **14**, 979-982, (2017).
12. Zhou, Y. *et al.* Metascape provides a biologist-oriented resource for the analysis of systems-level datasets. *Nature communications* **10**, 1523, (2019).
13. Korsunsky, I. *et al.* Fast, sensitive and accurate integration of single-cell data with Harmony. *Nat Methods* **16**, 1289-1296, (2019).

**Figure Legend of Supplementary Figures**

**Figure. S1 | Comparison of cell type annotation between different datasets and stages. A.** Overview of study flow. **B-C.** Hierarchical clustering analysis of cell types in representative tissues of heart (B) and kidney (C) between MCA and Tabula Muris Senis (TM). Color differences in branches of the dendrogram indicate major cell types. Color differences in the text of cell types indicate two databases. And the values in scale bar corresponding to branches of the dendrogram indicate correlation of cell types. **D-E.** Hierarchical clustering analysis of cell types in representative tissues of heart (D) and lung (E) across four mouse developmental stages. Color differences in branches of the dendrogram indicate major cell types marked by text. Solid dots of branch indicate each cell types and corresponding colors indicate different stages.

**Figure. S2 | Construction of mouse stromal cell landscape spanning seven developmental stages. A.** t-SNE plots showing total clusters of merged data without pre-cleared; Cells are colored by cell-type clusters. **B.** t-SNE plot of cells that were included and excluded from the analysis based on expression of marker genes for stromal cell and other cell types. Red indicates stromal cells; gray indicates excluded cells. **C.** Relative expression of a representative stromal cell gene (*Col1a2*) in each cluster. Color scale: dark green, low expression; light green, high expression. **D.** Bar graphs showing the number of analyzed stromal cells per stage after filtered. **E.** Heatmap showing the expression of the top 3 marker genes in each cluster. Color scale: red, high expression; blue, low expression. **F.** Relative abundance of each cluster in every tissue. The bubbles indicate the contributions of cells from each tissue to a cluster.

**Figure. S3 | Exploring heterogeneity of stromal cell types in mSCL. A-D.** Gene ontology (GO) analysis showing biological functions of the representative stromal cell subpopulations identified in mSCL. A. a common mesenchymal cell in embryos; B. a cell type of myofibroblast shared by tissues of placenta, ovary, uterus; C. Postn+ fibroblast both in heart and liver; D. Nrep+ fibroblast in lung tissue. The red color marks the go terms related to close biological functions of corresponding tissue. **E-G.** Heatmaps showing average relative gene expression in each subcluster in mSCL

in the following categories. E. Collagen & fibrillin genes; F. ECM associated genes; G. Cytokines & chemokines. **H.** Heatmap showing the relative expression of the top-ranked 2 enriched genes in each tissue. Color scale: red, high expression; blue, low expression.

**Figure. S4 | Construction of human stromal cell landscape including fetal- and adult- stages. A.** *t*-SNE plots showing total clusters of merged data without pre-cleared; Cells are colored by cell-type clusters. **B.** *t*-SNE plot of cells that were included and excluded from the analysis based on expression of marker genes for stromal cell and other cell types. Red indicates stromal cells; gray indicates excluded cells. **C.** Relative expression of a representative stromal cell gene (Col1a2) in each cluster. Color scale: dark green, low expression; light green, high expression. **D.** *t*-SNE plot of stromal cell with annotation information in human. Cells are colored by cell-type cluster. **E.** Heatmap showing the expression of the top 3 marker genes in each cluster. Color scale: red, high expression; blue, low expression.

**Figure. S5 | Dynamic cellular transition of mouse stromal cell during mouse development. A-B.** UMAP showing stromal cells with annotation information in mouse development. Cells are separately colored by stage information (A) and by cell-type cluster (B). **C-D.** Analysis of stromal cell trajectory by Monocle2. Cells are separately colored by stage information (C) and the predicted pseudotime (D). Color scale: dark, early time; light, late time. **E.** Dynamic distribution of each cell type in mouse stromal cell landscape along with the pseudotime trajectory. **F.** Analyzing stromal cell trajectory in heart by Monocle2. Cells are separately colored by stage information (upper panel) and the predicted pseudotime (lower panel). Color scale: dark, early time; light, late time. **G.** Heatmap showing the gene expression dynamics of stromal cells in heart development. Genes (row) are clustered and cells (column) are ordered according to the pseudotime trajectory. The top 300 genes in each cluster were analyzed with Gene ontology (GO), and represent GO terms are displayed.

**Figure. S6 | Dynamic gene signature of mouse stromal cell development. A-B**. Expression of ECM associated genes (A) and proliferation related genes (B) across 7 developmental stages. Circle sizes denote percentages of cells from

each stage, and color encodes average expression across all cells within stages. **C.** Violin plots showing the expression level of represent fetal-specific genes in stromal cell of different stages. *H19*, *Dlk1*, *Cdkn1c*, and *Mest* belong imprinted genes; *Crabp1* encodes a retinoic acid-binding protein 1. **D.** Bar graph displaying *Dlk1* expression mainly in stromal cells of fetal stages in total DMCA database. **E.** Expression of representative fetal-specific and adult-specific genes in human stromal cell landscape. Circle sizes denote percentages of cells from each stage, and color encodes average expression across all cells within stages. **F.** Gene ontology (GO) analysis of different expressed genes in fetal and adult stages corresponding E. The top GO terms are displayed and colored text represent the focused GO terms. **G.** Violin plots show the expression level of represent fetal-specific genes in each cluster of the mouse perturb-state atlas. Among the displayed genes, *Col1a2* acts as marker gene of fibroblast identity. The row represents the original clusters in the published article. The red label represents perturbation-specific cluster, which is also our main focus. **H.** Violin plots show the expression level of represent fetal-specific genes in each cluster of steady-state mouse fibroblast. Among the displayed genes, *Col1a2* acts as marker gene of fibroblast identity. The row represents the original clusters in the published article.

**Figure. S7 | Comparative analysis of perturbation-specific *Lrrc15*+ cluster in published perturb-state fibroblast atlas with cell types in mSCL. A.** Comparison of *Lrrc15*+ cluster in perturb-state atlas with mouse stromal cell landscape. Each row represents one cell type in our reference. Each column represents a cell from *Lrrc15*+ cluster. Pearson correlation coefficient was used to evaluate cell-type gene expression similarity. Red indicates a high correlation; grey indicates a low correlation. The red texts marked stromal cells in P0 stage with high correlation to *Lrrc15*+ cluster. **B-C**. UMAP showing subclusters of the *Lrrc15*+ cluster in perturb-state fibroblast atlas. Cells are separately colored by cell-type cluster (B) and by perturb-state information (C). The red texts marked perturb state of the focused subcluster. **D-F.** Featureplot displaying relative expression of a representative fetal gene (*H19*, *Cdkn1c*, and *Crabp1*) in each subcluster. Color scale: dark green, low expression; light green, high expression. **G-H.** UMAP visualization of integrated single-cell RNA-sequencing profiles of cells

from perturbation-specific *Lrrc15*+ cluster and mSCL. Cells are separately colored by merged cell-type cluster (G) and by mouse developmental-stage information (H). the circled region indicates closely correlated cells from the two data.

**Table S1. Comparative analysis of cell annotation of individual tissue from different single-cell data**

**Table S2. Differentially expressed genes detected in 18 stromal cell types from mSCL**

**Table S3. Differentially expressed genes detected in 16 stromal cell types from hSCL**

**Table S4. Cross-species comparison of stromal cells between human and mouse**

Figure S1

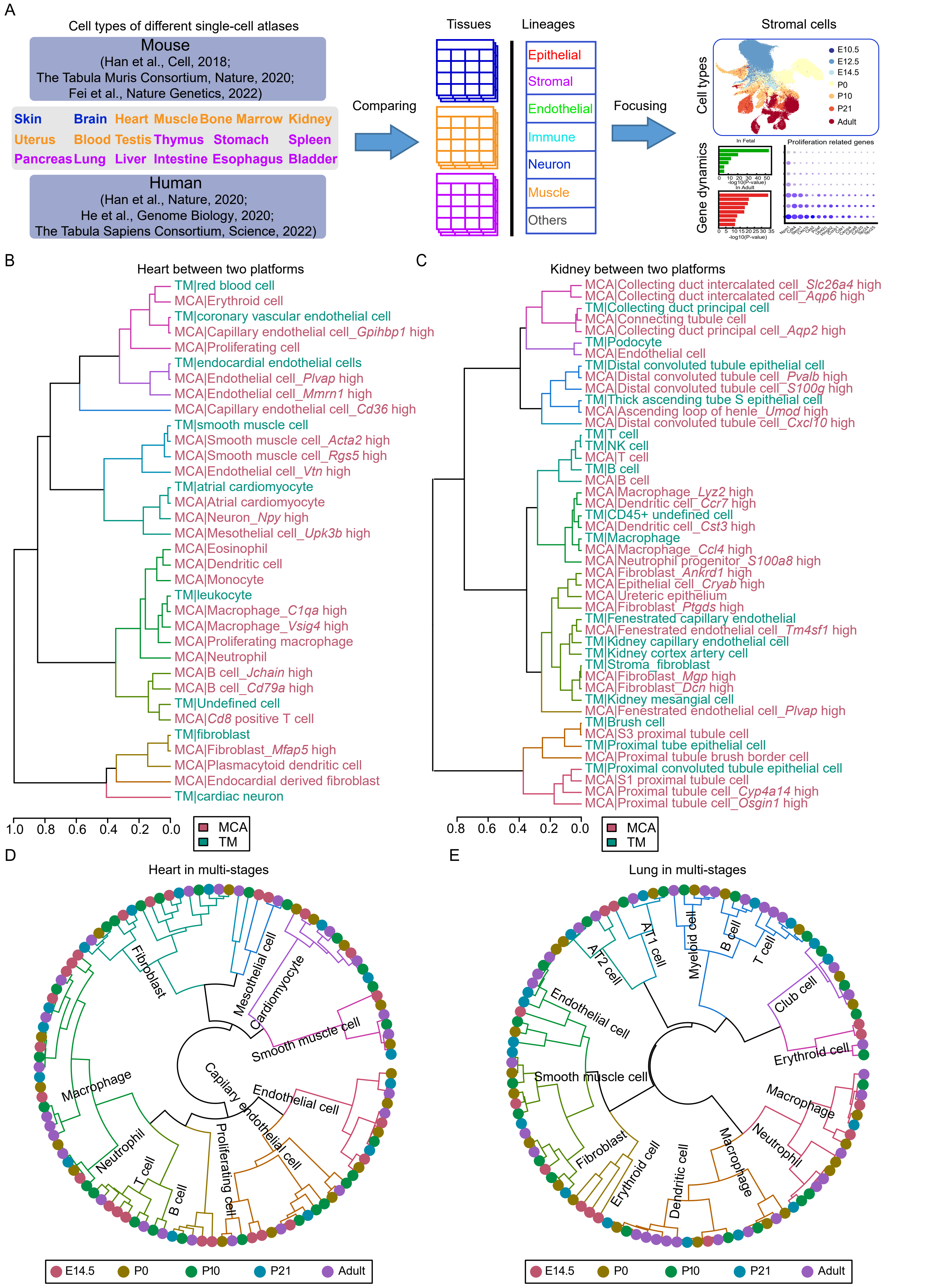
A
Cell types of different single-cell atlases
Mouse
(Han et al., Cell, 2018;
The Tabula Muris Consortium, Nature, 2020;
Fei et al., Nature Genetics, 2022)
Skin Brain Heart Muscle Bone Marrow Kidney
Uterus Blood Testis Thymus Stomach Spleen
Pancreas Lung Liver Intestine Esophagus Bladder
Human
(Han et al., Nature, 2020;
He et al., Genome Biology, 2020;
The Tabula Sapiens Consortium, Science, 2022)
Comparing
Tissues
Lineages
Epithelial
Stromal
Endothelial
Immune
Neuron
Muscle
Others
Focusing
Stromal cells
Cell types
E10.5
E12.5
E14.5
P0
P10
P21
Adult
Gene dynamics
In Fetal
In Adult
-log10(P-value)
Proliferation related genes
B
Heart between two platforms
TM|red blood cell
MCA|Erythroid cell
TM|coronary vascular endothelial cell
MCA|Capillary endothelial cell_Gpihbp1 high
MCA|Proliferating cell
TM|endocardial endothelial cells
MCA|Endothelial cell_Plvap high
MCA|Endothelial cell_Mmrn1 high
MCA|Capillary endothelial cell_Cd36 high
TM|smooth muscle cell
MCA|Smooth muscle cell_Acta2 high
MCA|Smooth muscle cell_Rgs5 high
MCA|Endothelial cell_Vtn high
TM|atrial cardiomyocyte
MCA|Atrial cardiomyocyte
MCA|Neuron_Npy high
MCA|Mesothelial cell_Upk3b high
MCA|Eosinophil
MCA|Dendritic cell
MCA|Monocyte
TM|leukocyte
MCA|Macrophage_C1qa high
MCA|Macrophage_Vsig4 high
MCA|Proliferating macrophage
MCA|Neutrophil
MCA|B cell_Jchain high
MCA|B cell_Cd79a high
TM|Undefined cell
MCA|Cd8 positive T cell
TM|fibroblast
MCA|Fibroblast_Mfap5 high
MCA|Plasmacytoid dendritic cell
MCA|Endocardial derived fibroblast
TM|cardiac neuron
1.0 0.8 0.6 0.4 0.2 0.0
MCA
TM
C
Kidney between two platforms
MCA|Collecting duct intercalated cell_Slc26a4 high
MCA|Collecting duct intercalated cell_Aqp6 high
TM|Collecting duct principal cell
MCA|Connecting tubule cell
MCA|Collecting duct principal cell_Aqp2 high
TM|Podocyte
MCA|Endothelial cell
TM|Distal convoluted tubule epithelial cell
MCA|Distal convoluted tubule cell_Pvalb high
MCA|Distal convoluted tubule cell_S100g high
TM|Thick ascending tube S epithelial cell
MCA|Ascending loop of henle_Umod high
MCA|Distal convoluted tubule cell_Cxcl10 high
TM|T cell
TM|NK cell
MCA|T cell
TM|B cell
MCA|B cell
MCA|Macrophage_Lyz2 high
MCA|Dendritic cell_Ccr7 high
TM|CD45+ undefined cell
MCA|Dendritic cell_Cst3 high
TM|Macrophage
MCA|Macrophage_Ccl4 high
MCA|Neutrophil progenitor_S100a8 high
MCA|Fibroblast_Ankrd1 high
MCA|Epithelial cell_Cryab high
MCA|Ureteric epithelium
MCA|Fibroblast_Ptgds high
TM|Fenestrated capillary endothelial
MCA|Fenestrated endothelial cell_Tm4sf1 high
TM|Kidney capillary endothelial cell
TM|Kidney cortex artery cell
TM|Stroma_fibroblast
MCA|Fibroblast_Mgp high
MCA|Fibroblast_Dcn high
TM|Kidney mesangial cell
MCA|Fenestrated endothelial cell_Plvap high
TM|Brush cell
MCA|S3 proximal tubule cell
TM|Proximal tube epithelial cell
MCA|Proximal tubule brush border cell
TM|Proximal convoluted tubule epithelial cell
MCA|S1 proximal tubule cell
MCA|Proximal tubule cell_Cyp4a14 high
MCA|Proximal tubule cell_Osgin1 high
0.8 0.6 0.4 0.2 0.0
MCA
TM
D
Heart in multi-stages
Fibroblast
Mesothelial cell
Cardiomyocyte
Smooth muscle cell
Capilary endothelial cell
Endothelial cell
Proliferating cell
Macrophage
Neutrophil
T cell
B cell
E14.5 P0 P10 P21 Adult
E
Lung in multi-stages
AT1 cell
AT2 cell
Myeloid cell
B cell
T cell
Club cell
Endothelial cell
Erythroid cell
Smooth muscle cell
Macrophage
Neutrophil
Fibroblast
Erythroid cell
Dendritic cell
Macrophage
E14.5 P0 P10 P21 Adult

Figure S2

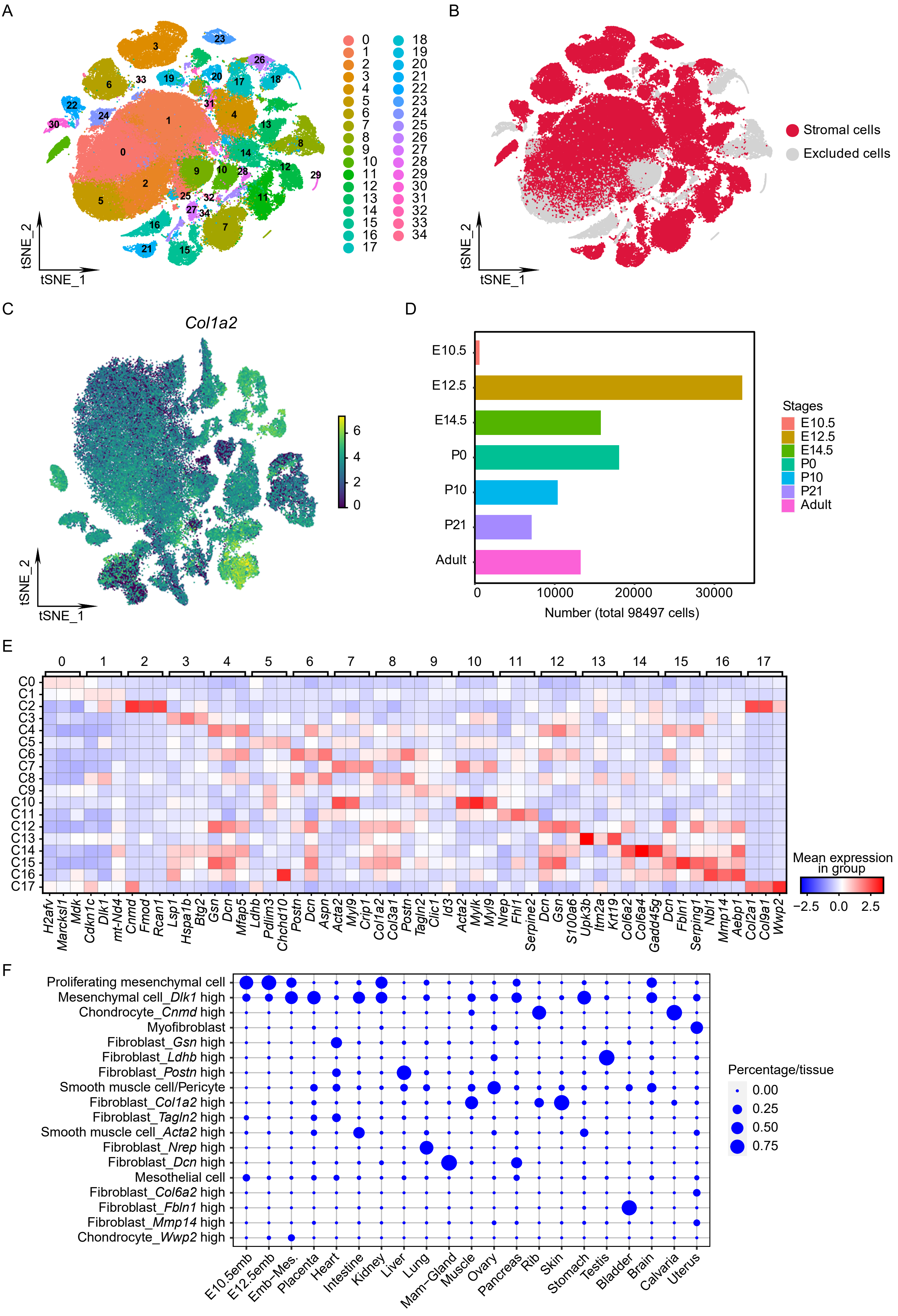

A
B
Stromal cells
Excluded cells
tSNE_1
tSNE_2
C
Col1a2
D
E10.5
E12.5
E14.5
P0
P10
P21
Adult
Stages
Number (total 98497 cells)
0
10000
20000
30000
E
Mean expression in group
-2.5 0.0 2.5
C0 C1 C2 C3 C4 C5 C6 C7 C8 C9 C10 C11 C12 C13 C14 C15 C16 C17
H2afv Marcksl1 Mdk Cdkn1c Dlk1 mt-Nd4 Cnmd Fmod Rcan1 Lsp1 Hspa1b Btg2 Gsn Dcn Mfap5 Ldhb Pdlim3 Chchd10 Postn Dcn Aspn Acta2 Myl9 Crip1 Col1a2 Col3a1 Postn Tagln2 Clic1 Id3 Acta2 Mylk Myl9 Nrep Fhl1 Serpine2 Dcn Gsn S100a6 Upk3b Itm2a Krt19 Col6a2 Col6a4 Gadd45g Dcn Fbln1 Serping1 Nbl1 Mmp14 Aebp1 Col2a1 Col9a1 Wwp2
F
Proliferating mesenchymal cell
Mesenchymal cell_Dlk1 high
Chondrocyte_Cnmd high
Myofibroblast
Fibroblast_Gsn high
Fibroblast_Ldhb high
Fibroblast_Postn high
Smooth muscle cell/Pericyte
Fibroblast_Col1a2 high
Fibroblast_Tagln2 high
Smooth muscle cell_Acta2 high
Fibroblast_Nrep high
Fibroblast_Dcn high
Mesothelial cell
Fibroblast_Col6a2 high
Fibroblast_Fbln1 high
Fibroblast_Mmp14 high
Chondrocyte_Wwp2 high
E10.5emb E12.5emb Emb-Mes. Placenta Heart Intestine Kidney Liver Lung Mam-Gland Muscle Ovary Pancreas Rib Skin Stomach Testis Bladder Brain Calvaria Uterus
Percentage/tissue
0.00
0.25
0.50
0.75

Figure S3

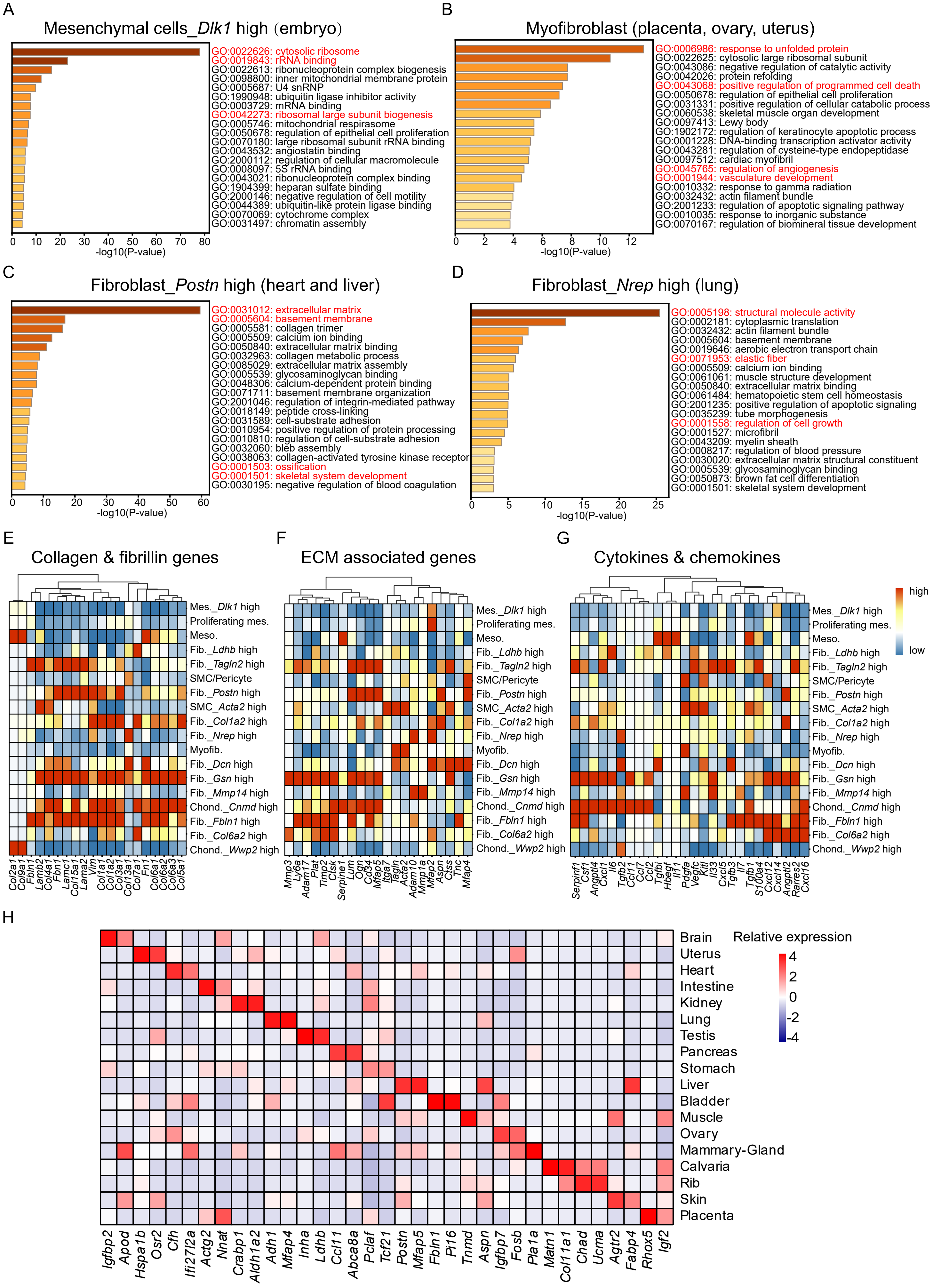

A
Mesenchymal cells_Dlk1 high (embryo)
GO:0022626: cytosolic ribosome
GO:0019843: rRNA binding
GO:0022613: ribonucleoprotein complex biogenesis
GO:0098800: inner mitochondrial membrane protein
GO:0005687: U4 snRNP
GO:1990948: ubiquitin ligase inhibitor activity
GO:0003729: mRNA binding
GO:0042273: ribosomal large subunit biogenesis
GO:0005746: mitochondrial respirasome
GO:0050678: regulation of epithelial cell proliferation
GO:0070180: large ribosomal subunit rRNA binding
GO:0043532: angiostatin binding
GO:2000112: regulation of cellular macromolecule
GO:0008097: 5S rRNA binding
GO:0043021: ribonucleoprotein complex binding
GO:1904399: heparan sulfate binding
GO:2000146: negative regulation of cell motility
GO:0044389: ubiquitin-like protein ligase binding
GO:0070069: cytochrome complex
GO:0031497: chromatin assembly
-log10(P-value)
B
Myofibroblast (placenta, ovary, uterus)
GO:0006986: response to unfolded protein
GO:0022625: cytosolic large ribosomal subunit
GO:0043086: negative regulation of catalytic activity
GO:0042026: protein refolding
GO:0043068: positive regulation of programmed cell death
GO:0050678: regulation of epithelial cell proliferation
GO:0031331: positive regulation of cellular catabolic process
GO:0060538: skeletal muscle organ development
GO:0097413: Lewy body
GO:1902172: regulation of keratinocyte apoptotic process
GO:0001228: DNA-binding transcription activator activity
GO:2000116: regulation of cysteine-type endopeptidase
GO:0097512: cardiac myofibril
GO:0045765: regulation of angiogenesis
GO:0001944: vasculature development
GO:0010332: response to gamma radiation
GO:0032432: actin filament bundle
GO:2001233: regulation of apoptotic signaling pathway
GO:0010035: response to inorganic substance
GO:0070167: regulation of biomineral tissue development
-log10(P-value)
C
Fibroblast_Postn high (heart and liver)
GO:0031012: extracellular matrix
GO:0005604: basement membrane
GO:0005581: collagen trimer
GO:0005509: calcium ion binding
GO:0050840: extracellular matrix binding
GO:0032963: collagen metabolic process
GO:0085029: extracellular matrix assembly
GO:0005539: glycosaminoglycan binding
GO:0048306: calcium-dependent protein binding
GO:0071711: basement membrane organization
GO:2001046: regulation of integrin-mediated pathway
GO:0018149: peptide cross-linking
GO:0031589: cell-substrate adhesion
GO:0010954: positive regulation of protein processing
GO:0010810: regulation of cell-substrate adhesion
GO:0032060: bleb assembly
GO:0038063: collagen-activated tyrosine kinase receptor
GO:0001503: ossification
GO:0001501: skeletal system development
GO:0030195: negative regulation of blood coagulation
-log10(P-value)
D
Fibroblast_Nrep high (lung)
GO:0005198: structural molecule activity
GO:0002181: cytoplasmic translation
GO:0032432: actin filament bundle
GO:0005604: basement membrane
GO:0019646: aerobic electron transport chain
GO:0071953: elastic fiber
GO:0005509: calcium ion binding
GO:0061061: muscle structure development
GO:0050840: extracellular matrix binding
GO:0061484: hematopoietic stem cell homeostasis
GO:2001235: positive regulation of apoptotic signaling
GO:0035239: tube morphogenesis
GO:0001558: regulation of cell growth
GO:0001527: microfibril
GO:0043209: myelin sheath
GO:0008217: regulation of blood pressure
GO:0030020: extracellular matrix structural constituent
GO:0005539: glycosaminoglycan binding
GO:0050873: brown fat cell differentiation
GO:0001501: skeletal system development
-log10(P-value)
E
Collagen & fibrillin genes
F
ECM associated genes
G
Cytokines & chemokines
high
low
Mes._Dlk1 high
Proliferating mes.
Meso.
Fib._Ldhb high
Fib._Tagln2 high
SMC/Pericyte
Fib._Postn high
SMC_Acta2 high
Fib._Col1a2 high
Fib._Nrep high
Myofib.
Fib._Dcn high
Fib._Gsn high
Fib._Mmp14 high
Chond._Cnmd high
Fib._Fbln1 high
Fib._Col6a2 high
Chond._Wwp2 high
Col2a1 Col9a1 Fbln1 Lamb2 Col4a1 Fbn1 Lamc1 Col15a1 Lama2 Vim Col1a1 Col1a2 Col3a1 Col13a1 Col7a1 Fn1 Col6a1 Col6a2 Col6a3 Col5a1
Mmp3 Ly6a Adam17 Plat Timp2 Ctsk Serpine1 Lum Ogn Cd34 Mfap5 Itga7 Tagln Acta2 Adam10 Mmp1a Mfap2 Aspn Ctss Tnc Mfap4
Serpinf1 Csf1 Angptl4 Cxcl1 Il6 Tgfb2 Ccl11 Ccl7 Ccl2 Tgfbi Hbegf Il11 Pdgfa Vegfc Kitl Il33 Cxcl5 Tgfb3 Il7 Tgfb1 S100a4 Cxcl12 Cxcl14 Angptl2 Rarres2 Cxcl16
H
Relative expression
Brain
Uterus
Heart
Intestine
Kidney
Lung
Testis
Pancreas
Stomach
Liver
Bladder
Muscle
Ovary
Mammary-Gland
Calvaria
Rib
Skin
Placenta
Igfbp2 Apod Hspa1b Osr2 Cfh Ifi27l2a Actg2 Nnat Crabp1 Aldh1a2 Adh1 Mfap4 Inha Ldhb Ccl11 Abca8a Pclaf Tcf21 Postn Mfap5 Fbln1 Pi16 Tnmd Aspn Igfbp7 Fosb Pla1a Matn1 Col11a1 Chad Ucma Agtr2 Fabp4 Rhox5 Igf2

Figure S4

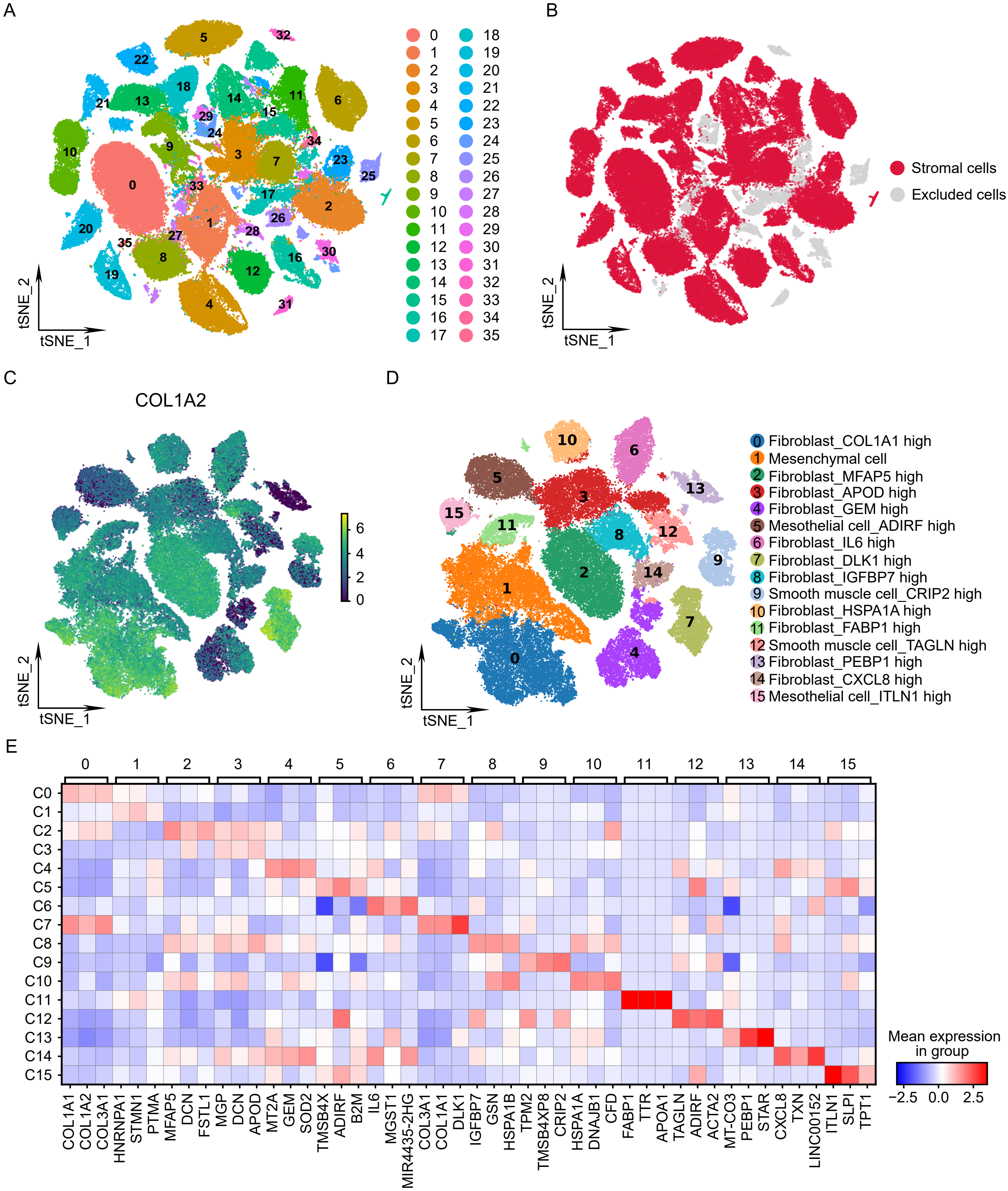
A
tSNE_1
tSNE_2
0 1 2 3 4 5 6 7 8 9 10 11 12 13 14 15 16 17 18 19 20 21 22 23 24 25 26 27 28 29 30 31 32 33 34 35
B
Stromal cells
Excluded cells
tSNE_1
tSNE_2
C
COL1A2
0 2 4 6
tSNE_1
tSNE_2
D
0 Fibroblast_COL1A1 high
1 Mesenchymal cell
2 Fibroblast_MFAP5 high
3 Fibroblast_APOD high
4 Fibroblast_GEM high
5 Mesothelial cell_ADIRF high
6 Fibroblast_IL6 high
7 Fibroblast_DLK1 high
8 Fibroblast_IGFBP7 high
9 Smooth muscle cell_CRIP2 high
10 Fibroblast_HSPA1A high
11 Fibroblast_FABP1 high
12 Smooth muscle cell_TAGLN high
13 Fibroblast_PEBP1 high
14 Fibroblast_CXCL8 high
15 Mesothelial cell_ITLN1 high
tSNE_1
tSNE_2
E
0 1 2 3 4 5 6 7 8 9 10 11 12 13 14 15
C0 C1 C2 C3 C4 C5 C6 C7 C8 C9 C10 C11 C12 C13 C14 C15
COL1A1 COL1A2 COL3A1 HNRNPA1 STMN1 PTMA MFAP5 DCN FSTL1 MGP DCN APOD MT2A GEM SOD2 TMSB4X ADIRF B2M IL6 MGST1 MIR4435-2HG COL3A1 COL1A1 DLK1 IGFBP7 GSN HSPA1B TPM2 TMSB4XP8 CRIP2 HSPA1A DNAJB1 CFD FABP1 TTR APOA1 TAGLN ADIRF ACTA2 MT-CO3 PEBP1 STAR CXCL8 TXN LINC00152 ITLN1 SLPI TPT1
Mean expression in group
−2.5 0.0 2.5

Figure S5

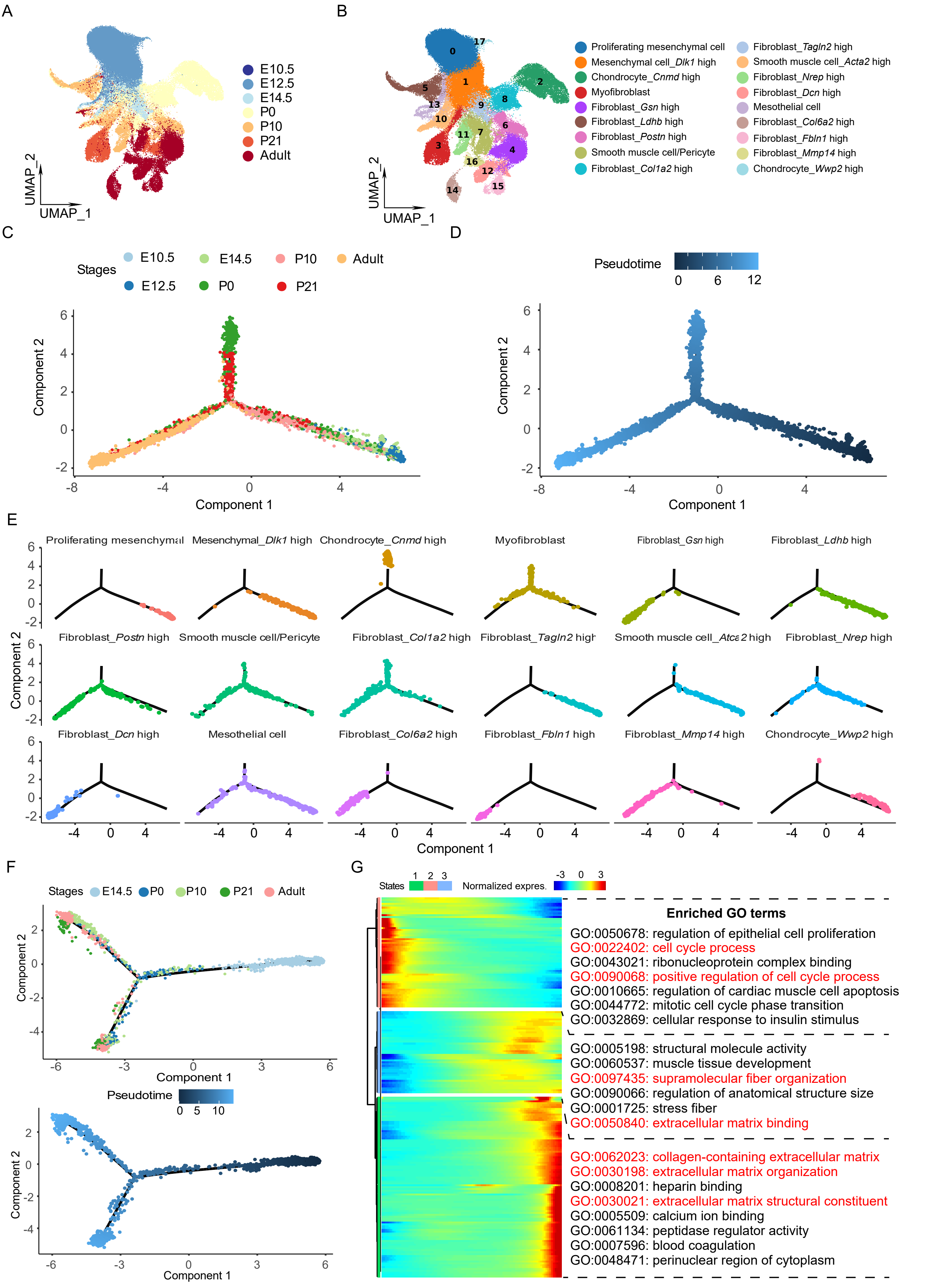

A
E10.5
E12.5
E14.5
P0
P10
P21
Adult
UMAP_2
UMAP_1
B
Proliferating mesenchymal cell
Mesenchymal cell_Dlk1 high
Chondrocyte_Cnmd high
Myofibroblast
Fibroblast_Gsn high
Fibroblast_Ldhb high
Fibroblast_Postn high
Smooth muscle cell/Pericyte
Fibroblast_Col1a2 high
Fibroblast_Tagln2 high
Smooth muscle cell_Acta2 high
Fibroblast_Nrep high
Fibroblast_Dcn high
Mesothelial cell
Fibroblast_Col6a2 high
Fibroblast_Fbln1 high
Fibroblast_Mmp14 high
Chondrocyte_Wwp2 high
C
Stages
Component 2
Component 1
D
Pseudotime
E
Proliferating mesenchymal
Mesenchymal_Dlk1 high
Chondrocyte_Cnmd high
Myofibroblast
Fibroblast_Gsn high
Fibroblast_Ldhb high
Fibroblast_Postn high
Smooth muscle cell/Pericyte
Fibroblast_Col1a2 high
Fibroblast_Tagln2 high
Smooth muscle cell_Atca2 high
Fibroblast_Nrep high
Fibroblast_Dcn high
Mesothelial cell
Fibroblast_Col6a2 high
Fibroblast_Fbln1 high
Fibroblast_Mmp14 high
Chondrocyte_Wwp2 high
F
G
States
Normalized expres.
Enriched GO terms
GO:0050678: regulation of epithelial cell proliferation
GO:0022402: cell cycle process
GO:0043021: ribonucleoprotein complex binding
GO:0090068: positive regulation of cell cycle process
GO:0010665: regulation of cardiac muscle cell apoptosis
GO:0044772: mitotic cell cycle phase transition
GO:0032869: cellular response to insulin stimulus
GO:0005198: structural molecule activity
GO:0060537: muscle tissue development
GO:0097435: supramolecular fiber organization
GO:0090066: regulation of anatomical structure size
GO:0001725: stress fiber
GO:0050840: extracellular matrix binding
GO:0062023: collagen-containing extracellular matrix
GO:0030198: extracellular matrix organization
GO:0008201: heparin binding
GO:0030021: extracellular matrix structural constituent
GO:0005509: calcium ion binding
GO:0061134: peptidase regulator activity
GO:0007596: blood coagulation
GO:0048471: perinuclear region of cytoplasm

Figure S6

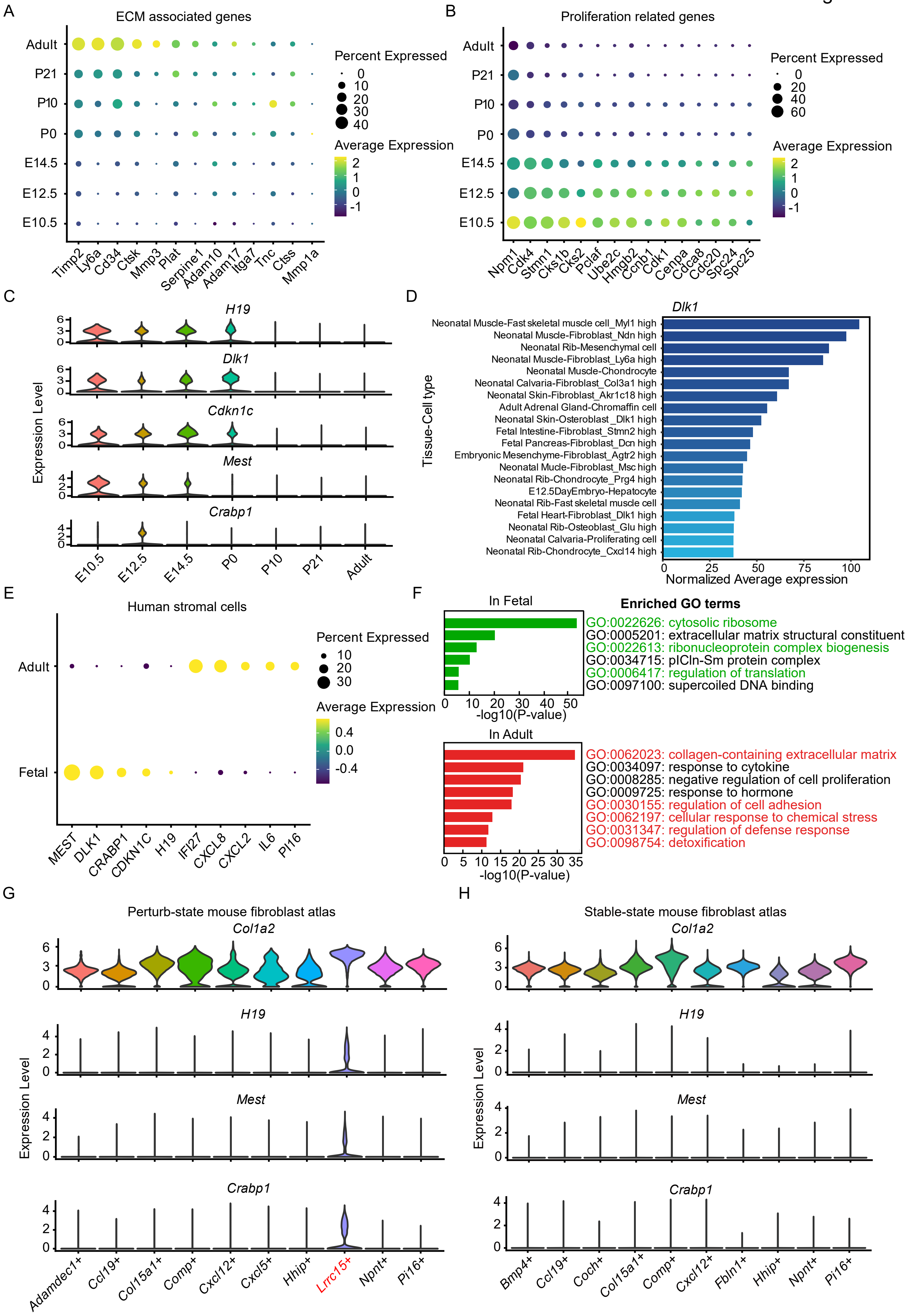
A
ECM associated genes
Adult
P21
P10
P0
E14.5
E12.5
E10.5
Timp2
Ly6a
Cd34
Ctsk
Mmp3
Plat
Serpine1
Adam10
Adam17
Itga7
Tnc
Ctss
Mmp1a
Percent Expressed
0
10
20
30
40
Average Expression
2
1
0
-1
B
Proliferation related genes
Npm1
Cdk4
Stmn1
Cks1b
Cks2
Pclaf
Ube2c
Hmgb2
Ccnb1
Cdk1
Cenpa
Cdca8
Cdc20
Spc24
Spc25
Percent Expressed
0
20
40
60
Average Expression
C
H19
Dlk1
Cdkn1c
Mest
Crabp1
Expression Level
E10.5
E12.5
E14.5
P0
P10
P21
Adult
D
Dlk1
Tissue-Cell type
Neonatal Muscle-Fast skeletal muscle cell_Myl1 high
Neonatal Muscle-Fibroblast_Ndn high
Neonatal Rib-Mesenchymal cell
Neonatal Muscle-Fibroblast_Ly6a high
Neonatal Muscle-Chondrocyte
Neonatal Calvaria-Fibroblast_Col3a1 high
Neonatal Skin-Fibroblast_Akr1c18 high
Adult Adrenal Gland-Chromaffin cell
Neonatal Skin-Osteroblast _Dlk1 high
Fetal Intestine-Fibroblast_Stmn2 high
Fetal Pancreas-Fibroblast_Dcn high
Embryonic Mesenchyme-Fibroblast_Agtr2 high
Neonatal Mucle-Fibroblast_Msc high
Neonatal Rib-Chondrocyte_Prg4 high
E12.5DayEmbryo-Hepatocyte
Neonatal Rib-Fast skeletal muscle cell
Fetal Heart-Fibroblast_Dlk1 high
Neonatal Rib-Osteoblast_Glu high
Neonatal Calvaria-Proliferating cell
Neonatal Rib-Chondrocyte_Cxcl14 high
0
25
50
75
100
Normalized Average expression
E
Human stromal cells
Adult
Fetal
MEST
DLK1
CRABP1
CDKN1C
H19
IFI27
CXCL8
CXCL2
IL6
PI16
Percent Expressed
10
20
30
Average Expression
0.4
0.0
-0.4
F
In Fetal
Enriched GO terms
GO:0022626: cytosolic ribosome
GO:0005201: extracellular matrix structural constituent
GO:0022613: ribonucleoprotein complex biogenesis
GO:0034715: pICln-Sm protein complex
GO:0006417: regulation of translation
GO:0097100: supercoiled DNA binding
-log10(P-value)
In Adult
GO:0062023: collagen-containing extracellular matrix
GO:0034097: response to cytokine
GO:0008285: negative regulation of cell proliferation
GO:0009725: response to hormone
GO:0030155: regulation of cell adhesion
GO:0062197: cellular response to chemical stress
GO:0031347: regulation of defense response
GO:0098754: detoxification
-log10(P-value)
G
Perturb-state mouse fibroblast atlas
Col1a2
H19
Mest
Crabp1
Expression Level
Adamdec1+
Ccl19+
Col15a1+
Comp+
Cxcl12+
Cxcl5+
Hhip+
Lrrc15+
Npnt+
Pi16+
H
Stable-state mouse fibroblast atlas
Col1a2
H19
Mest
Crabp1
Expression Level
Bmp4+
Ccl19+
Coch+
Col15a1+
Comp+
Cxcl12+
Fbln1+
Hhip+
Npnt+
Pi16+

Figure S7

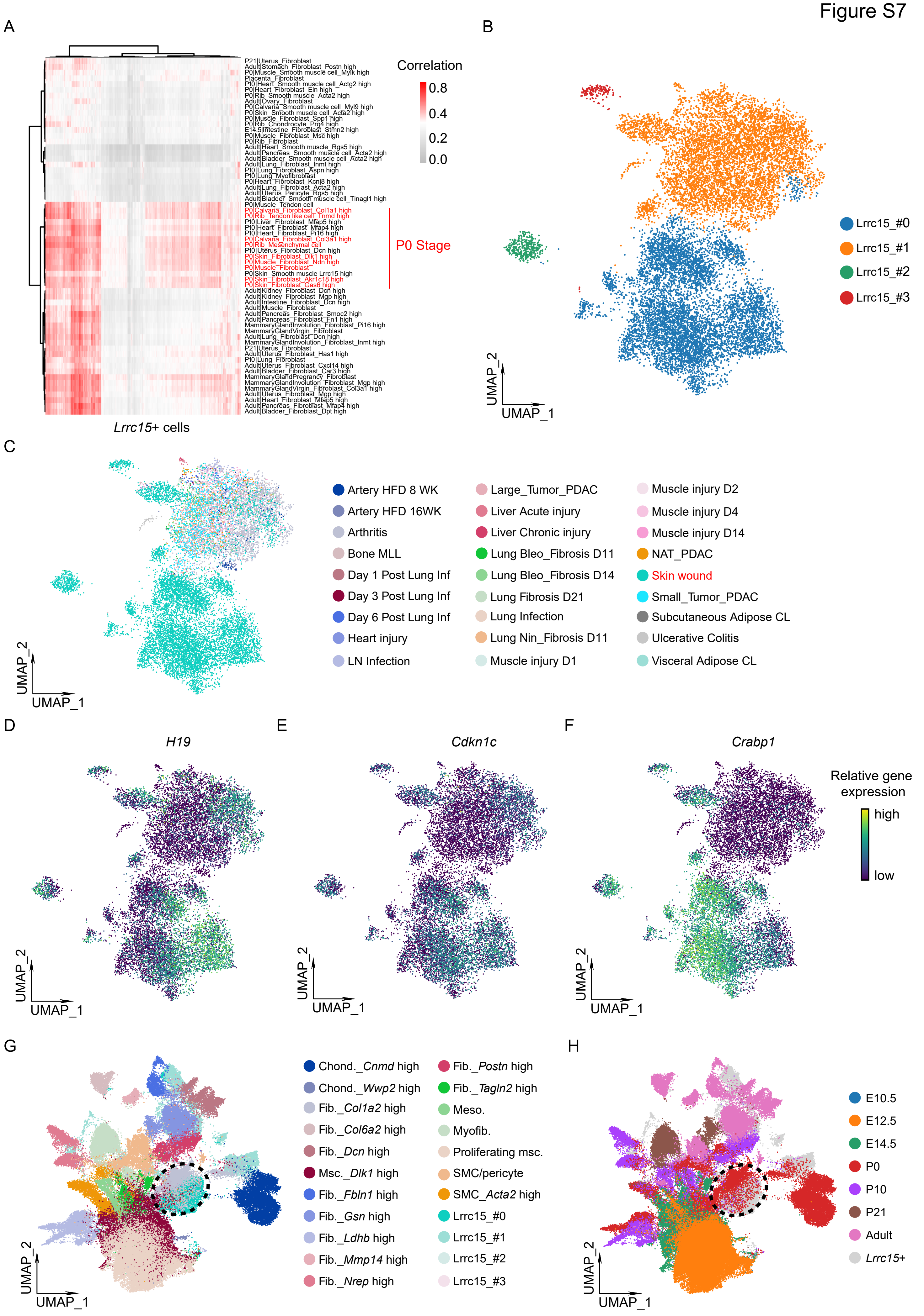
A
B
P21|Uterus_Fibroblast
Adult|Stomach_Fibroblast_Postn high
P0|Muscle_Smooth muscle cell_Mylk high
Placenta_Fibroblast
P10|Heart_Smooth muscle cell_Actg2 high
P0|Heart_Fibroblast_Eln high
P0|Rib_Smooth muscle_Acta2 high
Adult|Ovary_Fibroblast
P0|Calvaria_Smooth muscle cell_Myl9 high
P0|Skin_Smooth muscle cell_Acta2 high
P0|Muscle_Fibroblast_Spp1 high
P0|Rib_Chondrocyte_Prg4 high
E14.5|Intestine_Fibroblast_Stmn2 high
P0|Muscle_Fibroblast_Msc high
P0|Rib_Fibroblast
Adult|Heart_Smooth muscle_Rgs5 high
Adult|Pancreas_Smooth muscle cell_Acta2 high
Adult|Bladder_Smooth muscle cell_Acta2 high
Adult|Lung_Fibroblast_Inmt high
P10|Lung_Fibroblast_Aspn high
P10|Lung_Myofibroblast
P0|Heart_Fibroblast_Kcnj8 high
Adult|Lung_Fibroblast_Acta2 high
Adult|Uterus_Pericyte_Rgs5 high
Adult|Bladder_Smooth muscle cell_Tinagl1 high
P0|Muscle_Tendon cell
P0|Calvaria_Fibroblast_Col1a1 high
P0|Rib_Tendon like cell_Tnmd high
P10|Liver_Fibroblast_Mfap5 high
P10|Heart_Fibroblast_Mfap4 high
P10|Heart_Fibroblast_Pi16 high
P0|Calvaria_Fibroblast_Col3a1 high
P0|Rib_Mesenchymal cell
P10|Uterus_Fibroblast_Dcn high
P0|Skin_Fibroblast_Dlk1 high
P0|Muscle_Fibroblast_Ndn high
P0|Muscle_Fibroblast
P0|Skin_Smooth muscle Lrrc15 high
P0|Skin_Fibroblast_Akr1c18 high
P0|Skin_Fibroblast_Gas6 high
Adult|Kidney_Fibroblast_Dcn high
Adult|Kidney_Fibroblast_Mgp high
Adult|Intestine_Fibroblast_Dcn high
Adult|Muscle_Fibroblast
Adult|Pancreas_Fibroblast_Smoc2 high
Adult|Pancreas_Fibroblast_Fn1 high
MammaryGlandInvolution_Fibroblast_Pi16 high
MammaryGlandVirgin_Fibroblast
Adult|Lung_Fibroblast_Dcn high
MammaryGlandInvolution_Fibroblast_Inmt high
P21|Uterus_Fibroblast
Adult|Uterus_Fibroblast_Has1 high
P10|Lung_Fibroblast
Adult|Uterus_Fibroblast_Cxcl14 high
Adult|Bladder_Fibroblast_Car3 high
MammaryGlandPregrancy_Fibroblast
MammaryGlandInvolution_Fibroblast_Mgp high
MammaryGlandVirgin_Fibroblast_Col3a1 high
Adult|Uterus_Fibroblast_Mgp high
Adult|Heart_Fibroblast_Mfap5 high
Adult|Pancreas_Fibroblast_Mfap4 high
Adult|Bladder_Fibroblast_Dpt high
Correlation
0.8
0.4
0.2
0.0
P0 Stage
Lrrc15+ cells
Lrrc15_#0
Lrrc15_#1
Lrrc15_#2
Lrrc15_#3
UMAP_2
UMAP_1
C
Artery HFD 8 WK
Artery HFD 16WK
Arthritis
Bone MLL
Day 1 Post Lung Inf
Day 3 Post Lung Inf
Day 6 Post Lung Inf
Heart injury
LN Infection
Large_Tumor_PDAC
Liver Acute injury
Liver Chronic injury
Lung Bleo_Fibrosis D11
Lung Bleo_Fibrosis D14
Lung Fibrosis D21
Lung Infection
Lung Nin_Fibrosis D11
Muscle injury D1
Muscle injury D2
Muscle injury D4
Muscle injury D14
NAT_PDAC
Skin wound
Small_Tumor_PDAC
Subcutaneous Adipose CL
Ulcerative Colitis
Visceral Adipose CL
UMAP_2
UMAP_1
D
H19
E
Cdkn1c
F
Crabp1
Relative gene expression
high
low
UMAP_2
UMAP_1
G
Chond._Cnmd high
Chond._Wwp2 high
Fib._Col1a2 high
Fib._Col6a2 high
Fib._Dcn high
Msc._Dlk1 high
Fib._Fbln1 high
Fib._Gsn high
Fib._Ldhb high
Fib._Mmp14 high
Fib._Nrep high
Fib._Postn high
Fib._Tagln2 high
Meso.
Myofib.
Proliferating msc.
SMC/pericyte
SMC_Acta2 high
Lrrc15_#0
Lrrc15_#1
Lrrc15_#2
Lrrc15_#3
UMAP_2
UMAP_1
H
E10.5
E12.5
E14.5
P0
P10
P21
Adult
Lrrc15+
UMAP_2
UMAP_1
